# Supplementary material for: Longitudinal association between self-rated health and psychological well-being in a sample of Spanish university graduates
Source: PLoS One. 2025 Dec 26;20(12):e0338320. doi: 10.1371/journal.pone.0338320 (PMC12742725; doi:10.1371/journal.pone.0338320)
Supplement: S1 Table — (DOCX) [file pone.0338320.s001.docx]

## Longitudinal association between self-rated health and psychological well-being in a sample of Spanish university graduates

Supplementary Table 1. Multivariable-adjusted beta coefficients and 95% confidence intervals for associations between baseline self-rated health and psychological well-being after 14 years of follow-up (n=2,927, SUN cohort)

|  | Fair or poor  (1-2) | Good  (3) | Very good  (4) | Excellent  (5) | p for trend |
| --- | --- | --- | --- | --- | --- |
| N | 119 | 1 336 | 1 201 | 271 |  |
| Overall psychological well-being mean(SD) | 127.30 (23.7) | 136.10 (17.2) | 141.84 (15.8) | 145.13 (17.2) |  |
| Crude | 0 (ref.) | 8.8 (5.6,11.9) | 14.5 (11.3,17.7) | 17.8 (14.1,21.4) | <0.001 |
| Model 1 ^a^ | 0 (ref.) | 8.4 (5.3,11.6) | 13.9 (10.7,17.2) | 17.2 (13.5,20.9) | <0.001 |
| Model 2 ^b^ | 0 (ref.) | 8.2 (5.0,11.4) | 13.4 (10.2,16.7) | 16.4 (12.7,20.0) | <0.001 |
| Model 3 ^c^ | 0 (ref.) | 7.3 (4.1,10.5) | 12.3 (9.1,15.6) | 15.1 (11.4,18.8) | <0.001 |
| Self-acceptance (mean, SD) | 17.4 | 18.9 | 19.9 | 20.3 |  |
| Crude | 0 (ref.) | 1.5 (0.9,2.0) | 2.4 (1.8,2.9) | 2.8 (2.2,3.4) | <0.001 |
| Model 1^a^ | 0 (ref.) | 1.4 (0.9,2.0) | 2.4 (1.8,2.9) | 2.8 (2.2,3.4) | <0.001 |
| Model 2^b^ | 0 (ref.) | 1.4 (0.9,2.0) | 2.3 (1.8,2.9) | 2.7 (2.1,3.4) | <0.001 |
| Model 3^c^ | 0 (ref.) | 1.3 (0.7,1.8) | 2.2 (1.6,2.7) | 2.5 (1.8,3.1) | <0.001 |
| Autonomy (mean, SD) | 25.74 | 26.79 | 27.72 | 28.08 |  |
| Crude | 0 (ref.) | 1.0 (0.1,1.9) | 1.9 (1.0,2.8) | 2.3 (1.3,3.3) | <0.001 |
| Model 1^a^ | 0 (ref.) | 0.9 (0.1,1.8) | 1.9 (1.0,2.8) | 2.2 (1.2,3.3) | <0.001 |
| Model 2^b^ | 0 (ref.) | 0.9 (0.05,1.8) | 1.8 (0.9,2.7) | 2.2 (1.1,3.2) | <0.001 |
| Model 3^c^ | 0 (ref.) | 0.7 (-0.1,1.6) | 1.6 (0.7,2.5) | 2.0 (0.9,3.0) | <0.001 |
| Positive relations with others (mean, SD) | 21.3 | 23.2 | 24.4 | 25.3 |  |
| Crude | 0 (ref.) | 1.9 (1.0,2.7) | 3.0 (2.2,3.9) | 4.0 (3.0,4.9) | <0.001 |
| Model 1 ^a^ | 0 (ref.) | 1.7 (0.9,2.6) | 2.7 (1.9,3.6) | 3.6 (2.7,4.6) | <0.001 |
| Model 2 ^b^ | 0 (ref.) | 1.7 (0.9,2.5) | 2.7 (1.8,3.5) | 3.5 (2.5,4.4) | <0.001 |
| Model 3 ^c^ | 0 (ref.) | 1.6 (0.8,2.4) | 2.5 (1.7,3.4) | 3.3 (2.3,4.3) | <0.001 |
| Environmental mastery (mean, SD) | 22.2 | 24.0 | 25.1 | 25.7 |  |
| Crude | 0 (ref.) | 1.7 (1.1,2.4) | 2.8 (2.1,3.5) | 3.5 (2.7,4.2) | <0.001 |
| Model 1^a^ | 0 (ref.) | 1.7 (1.0,2.4) | 2.8 (2.1,3.4) | 3.4 (2.6,4.2) | <0.001 |
| Model 2^b^ | 0 (ref.) | 1.7 (1.0,2.3) | 2.7 (2.0,3.3) | 3.3 (2.5,4.0) | <0.001 |
| Model 3^c^ | 0 (ref.) | 1.4 (0.8,2.1) | 2.4 (1.7,3.0) | 3.0 (2.2,3.7) | <0.001 |
| Purpose in life (mean, SD) | 22.4 | 23.7 | 24.7 | 25.4 |  |
| Crude | 0 (ref.) | 1.3 (0.6,2.0) | 2.3 (1.6,3.0) | 2.9 (2.1,3.7) | <0.001 |
| Model 1^a^ | 0 (ref.) | 1.2 (0.5,1.9) | 2.3 (1.6,3.0) | 2.9 (2.1,3.7) | <0.001 |
| Model 2^b^ | 0 (ref.) | 1.2 (0.5,1.9) | 2.2 (1.5,2.9) | 2.7 (1.9,3.5) | <0.001 |
| Model 3^c^ | 0 (ref.) | 1.0 (0.3,1.7) | 2.0 (1.3,2.7) | 2.5 (1.7,3.3) | <0.001 |
| Personal growth (mean, SD) | 17.8 | 19.0 | 19.7 | 20.0 |  |
| Crude | 0 (ref.) | 1.2 (0.6,1.8) | 1.8 (1.2,2.4) | 2.1 (1.5,2.8) | <0.001 |
| Model 1^a^ | 0 (ref.) | 1.1 (0.5,1.6) | 1.6 (1.0,2.2) | 1.9 (1.3,2.6) | <0.001 |
| Model 2^b^ | 0 (ref.) | 1.0 (0.5,1.6) | 1.5 (0.9,2.1) | 1.8 (1.1,2.4) | <0.001 |
| Model 3^c^ | 0 (ref.) | 1.1 (0.5,1.7) | 1.5 (1.0,2.1) | 1.8 (1.1,2.5) | <0.001 |

N: number of participants, SD: standard deviation

^a^ Adjusted for sex, age, level of education, marital status and number of children

^b^ Additionally adjusted for leisure time physical exercise, a score of total physical activity [1], energy intake, adherence to the Mediterranean diet, hours of sleep per day, smoking, lifetime tobacco exposure (pack-years), alcohol consumption, and the frequency of interaction in social networks

^c^ Additionally adjusted for prevalence of depression, diabetes, cardiovascular disease and cancer, and body mass index
