# Supplementary material for: Longitudinal association between self-rated health and psychological well-being in a sample of Spanish university graduates
Source: PLoS One. 2025 Dec 26;20(12):e0338320. doi: 10.1371/journal.pone.0338320 (PMC12742725; doi:10.1371/journal.pone.0338320)
Supplement: S2 Table — (DOCX) [file pone.0338320.s002.docx]

## Longitudinal association between self-rated health and psychological well-being in a sample of Spanish university graduates

Supplementary Table 2. Beta coefficients and 95% CI of overall psychological well-being and 6 dimensions according to the change in the SRH between the baseline and 4 years of follow up (N=2 740)

|  | Crude Model | Model 1 | Model 2 | Model 3 |
| --- | --- | --- | --- | --- |
| N | 2740 | 2740 | 2740 | 2740 |
| No change in SRH (n= 1 704) | 0 (ref.) | 0 (ref.) | 0 (ref.) | 0 (ref.) |
| Overall PWB |  |  |  |  |
| SRH worsened (n=540) | -3.0 (-4.8,-1.3) | -2.9 (-4.7,-1.2) | -2.7 (-4.4,-1.0) | -2.6 (-4.3,-0.9) |
| SRH improved  (n= 496) | 5.6 (3.9,7.3) | 5.4 (3.7,7.2) | 5.2 (3.5,6.9) | 5.1 (3.4,6.8) |
| Self-acceptance |  |  |  |  |
| SRH worsened | -0.7 (-1.0, -0.4) | -0.7 (-1.0, -0.4) | -0.7 (-0.9, -0.4) | -0.6 (-0.9, -0.3) |
| SRH improved | 0.08 (0.5, 1.1) | 0.8 (0.5, 1.1) | 0.8 (0.5, 1.0) | 0.8 (0.5, 1.1) |
| Autonomy |  |  |  |  |
| SRH worsened | -0.3 (-0.8, 0.2) | -0.3 (-0.8, 0.2) | -0.3 (-0.1, 0.2) | -0.2 (-0.7, 0.2) |
| SRH improved | 0.5 (0.01, 1.0) | 0.5 (0.1, 1.0) | 0.5 (0.04, 1.0) | 0.5 (0.03, 1.0) |
| Positive relations with others |  |  |  |  |
| SRH worsened | -0.5 (-1.0, -0.1) | -0.5 (-.9, -0.01) | -0.4 (-0.9, 0.01) | -0.4 (-0.9, 0.03) |
| SRH improved | 0.9 (0.5, 1.4) | 0.80(0.3, 1.3) | 0.7 (0.3, 1.2) | 0.7 (0.3, 1.2) |
| Environmental mastery |  |  |  |  |
| SRH worsened | -0.6 (-1.0, -0.3) | -0.6 (-1.0, -0.2) | -0.5 (-0.9, -0.2) | -0.5 (-0.9, -0.1) |
| SRH improved | 1.2 (0.9, 1.6) | 1.2 (0.9, 1.6) | 1.2 (0.8, 1.5) | 1.2 (0.8, 1.5) |
| Purpose in life |  |  |  |  |
| SRH worsened | -0.7 (-1.2, -0.3) | -0.7 (-1.1, -0.3) | -0.6 (-1.0, -0.3) | -0.6 (-1.0, -0.3) |
| SRH improved | 1.2 (0.9, 1.6) | 1.2 (0.9, 1.6) | 1.2 (0.8, 1.6) | 1.2 (0.8, 1.5) |
| Personal growth |  |  |  |  |
| SRH worsened | -0.2 (-0.5, 0.1) | -0.2 (-0.5, 0.1) | -0.1 (-0.4, 0.2) | -0.1 (-0.4, 0.2) |
| SRH improved | 0.7 (0.4, 1.0) | 0.6 (0.3, 0.9) | 0.6 (0.2, 0.9) | 0.6 (0.2, 0.9) |
