# Supplementary material for: Longitudinal association between self-rated health and psychological well-being in a sample of Spanish university graduates
Source: PLoS One. 2025 Dec 26;20(12):e0338320. doi: 10.1371/journal.pone.0338320 (PMC12742725; doi:10.1371/journal.pone.0338320)
Supplement: S3 Table — (DOCX) [file pone.0338320.s003.docx]

## Longitudinal association between self-rated health and psychological well-being in a sample of Spanish university graduates

Supplementary Table 3. Multivariable adjusted* odds ratio and 95% CI for above-median psychological well-being (and its six dimensions) after 14 years according to baseline self-rated health categories (SUN cohort)

|  | **Fair or poor** | **Good** | **Very good** | **Excellent** | **p for trend** |
| --- | --- | --- | --- | --- | --- |
| N | 119 | 1 336 | 1 201 | 271 |  |
| N with higher PWB (%) | 36 (2.5) | 554 (39.4) | 649 (46.2) | 167 (11.9) |  |
| Overall psychological well-being | 1 (ref.) | 1.4 (0.9, 2.2) | 2.2 (1.5, 3.4) | 3.0 (1.8, 4.8) | <0.001 |
| Self-acceptance | 1 (ref.) | 1.3 (0.8, 2.0) | 2.4 (1.5, 3.8) | 3.2 (1.9, 5.4) | <0.001 |
| Autonomy | 1 (ref.) | 1.3 (0.8, 1.9) | 1.7 (1.1, 2.6) | 2.2 (1.4, 3.6) | <0.001 |
| Positive relations with others | 1 (ref.) | 1.5 (0.9, 2.4) | 2.5 (1.5, 3.9) | 3.3 (2.0, 5.5) | <0.001 |
| Environmental mastery | 1 (ref.) | 1.3 (0.8, 2.0) | 2.3 (1.5, 3.6) | 2.9 (1.8, 4.8) | <0.001 |
| Purpose in life | 1 (ref.) | 0.8 (0.5, 1.3) | 1.4 (0.9, 2.1) | 1.9 (1.2, 3.0) | <0.001 |
| Personal growth | 1 (ref.) | 1.3 (0.8, 2.0) | 1.7 (1.1, 2.7) | 2.1 (1.3, 3.5) | <0.001 |

Adjusted for sex, age, level of education, marital status, number of children, leisure time physical exercise, a score of total physical activity, caloric intake, adherence to the Mediterranean diet, hours of sleep per day, smoking, lifetime tobacco exposure (pack-years), alcohol consumption, the frequency of interaction in social networks, prevalence of depression, diabetes, cardiovascular disease and cancer and body mass index

1. Alvarez-Alvarez I, Zazpe I, Pérez de Rojas J, Bes-Rastrollo M, Ruiz-Canela M, Fernandez-Montero A, et al. Mediterranean diet, physical activity and their combined effect on all-cause mortality: The Seguimiento Universidad de Navarra (SUN) cohort. Prev Med (Baltim). 2018;106: 45–52. doi:10.1016/J.YPMED.2017.09.021
