# Supplementary material for: Longitudinal association between self-rated health and psychological well-being in a sample of Spanish university graduates
Source: PLoS One. 2025 Dec 26;20(12):e0338320. doi: 10.1371/journal.pone.0338320 (PMC12742725; doi:10.1371/journal.pone.0338320)
Supplement: Attachment — Submitted filename: Review of Longitudinal association between self_250902.docx [file pone.0338320.s004.docx]

Review of Longitudinal association between self-rated health and psychological well-being in a sample of Spanish university graduates

Thank you for an interesting manuscript describing the association between PWB and SRH and the change of SRH within 4 years, and the association with PWB, using a large sample with a long follow-up.

Overall, it is well-written, but I have some small comments listed under each heading.

A major comment
All tables should be able to stand alone, with only the table text included. I think none of the tables can do so at the moment. Currently, I am unable to interpret the tables without going back to the method and statistics and reading once again what analysis you present the result of in each table.
I think you should choose one thing to analyze, either the change in SRH over the 4 years **or** the baseline SRH, to make it easier to interpret.

**Abstract**
Generally, I get a bit disturbed over the use of abbreviations within the abstract. Personally, I think it lowers the readability of the text. Please review to determine if abbreviations are necessary to avoid exceeding the word and character limitations.

The description of the participants' age is the age at baseline or at follow-up; please clarify.

**Introduction**

Row 65: Why do you have a secondary objective? Is there any reason to believe that SRH should change in 4 years? Please clarify what your basis is for this idea. You haven’t mentioned anything about a change in SRH over time or what events might influence it. I think there are some studies done describing changes in SRH over time, which could be good to incorporate.

**Methods**

Write out the abbreviation first time used, SUN, which I presume you mean Seguimiento Universidad de Navarra

Even if the study has been described elsewhere, I still want to know how long the inclusion of participants was ongoing. How many were included at baseline, proportion of men and women.

Was it decided at study start that the follow-up questionnaire should be filled out after 14 years?

If I understand it right, you included participants from 1999 and 2022? Or between which time points were the included participants recruited?

**Figure 1**.
What is included in pre-baseline variables? Was the pre-baseline 4 years before? What information is gathered from pre-baseline? I think you need to add some text to explain Figure 1to guide the reader. A figure or a table should be able to stand alone and be understood.

**Participants**

Row 85, Follow-up from which time point?

Row 86, which time point (t?) do you mean is the baseline in this context?

**Figure 2** is very nice and easy to understand.

Row 90, when did the participants receive the written information?

I am missing the age at which they were included in the study.

**Variables**

Rows 103- 105, I think you should rewrite these sentences to make it easier to understand. Especially the sentence starting with “It has been…”
For example,” The latter item served as an indicator of how frequently participants interacted within their social networks and has been widely applied in epidemiological studies examining associations …..…”

**Statistics**

Row 126, I am unfamiliar with the wording raw model, In previous papers, I think I have seen it mentioned as a crude model, but it may be the same, but different wording

You have used a lot of different variables in the multiple linear regression. Did you analyse whether they were correlated with each other?

I think that METS/ week and a score of total physical activity are strongly correlated and may not be proper to use both. The same goes for smoking habits and lifetime tobacco exposure

There appear to be several potential limitations in the statistical analysis, as various variables may act as mediators or confounders of the outcome. I cannot see that you have investigated VIF (Variance Inflation Factor) to detect multicollinearity in the regression models**.**

Why did you adjust for depression, diabetes, cardiovascular disease, and cancer instead of excluding them from the analysis, since it is well-known that a chronic disease affects SRH and the well-being, alternatively conduct a separate analysis for the group with chronic disease?

Due to the advanced statistical analysis, I would recommend that the analysis be reviewed by an expert in statistics.

Rows 155-160, it is nicely described how you have handled missing values. However, I did not think this was the right way; a more proper approach would be to exclude those who didn´t answer, just because you do not know the reason why they didn’t answer. I could be due to a lack of time. Smokers could be ashamed to admit that they are smokers and, because of this, skip answering the question, or have you thought about the method of carrying the last number forward? Regarding marital status, I think that was a proper way.

Please explain how you did the imputation in hours of sleep; the way you have done it seems risky and may affect the results.

**Results**

Row 164, follow-up not followed-up.

Table 1. There is a lack of information about what the numbers in the parentheses represent in several of the variables.

Row 182, I think it should be more proper to present the main findings of Table 2 and then refer to the table, instead of writing “Table 2 presents the….”

Table 2, first row (mean, SD) in parentheses, is it the mean or SD?

Row 211, see the comment above regarding row 182. And the table shows the result of the multiple linear regression stratified by…..

Row 211, since I haven't seen any explanation of whether the age refers to baseline, pre-baseline, or follow-up, it is hard to interpret

Tables 3, 4, and 5: What do the numbers within the parentheses represent?

**Discussion**

Row 267, If this is the particular novel finding I think this should be presented as the main finding. As you write in rows above, your findings just (mor or less) replicate previous findings.

Row 273, What kind of intervention do you imagine may affect the individual's perception of their health? Is it just their perceptions that should be changed? Or is it the actual physical health that needs improvement for a changed perception of one´s own health?

Row 282, is it really the subjective assessment that shapes the PWB over time? I think it is more complex. The measurement may indicate in which direction the PWB may take over time (increasing or decreasing)

Row 283- 287, Can you elaborate? It sounds like you mean that it is only the attitude to how you perceive your health that should change, and the actual physical and mental health, and further aspects do not affect the perception of one´s health.

How do you think your results should be used clinically? Yes, I understand that a holistic approach to health promotion and prevention is necessary, but in what specific way should I use it in my everyday work?
